# Structural Changes in Individual Retinal Layers in Diabetic Macular Edema

**DOI:** 10.1155/2013/920713

**Published:** 2013-08-29

**Authors:** Tomoaki Murakami, Nagahisa Yoshimura

**Affiliations:** Department of Ophthalmology and Visual Sciences, Kyoto University Graduate School of Medicine, 54 Shogoin-Kawaracho, Sakyo, Kyoto 606-8507, Japan

## Abstract

Optical coherence tomography (OCT) has enabled objective measurement of the total retinal thickness in diabetic macular edema (DME). The central retinal thickness is correlated modestly with visual impairment and changes paradoxically after treatments compared to the visual acuity. This suggests the clinical relevance of the central retinal thickness in DME and the presence of other factors that affect visual disturbance. Recent advances in spectral-domain (SD) OCT have provided better delineation of the structural changes and fine lesions in the individual retinal layers. Cystoid spaces in the inner nuclear layer and outer plexiform layer are related to quantitative and qualitative parameters in fluorescein angiography. OCT often shows vitreoretinal interface abnormalities in eyes with sponge-like retinal swelling. Serous retinal detachment is sometimes accompanied by hyperreflective foci in the subretinal fluid, which exacerbates the pathogenesis at the interface of the photoreceptors and retinal pigment epithelium. Photoreceptor damage at the fovea is thought to be represented by disruption of the external limiting membrane or the junction between the inner and outer segment lines and is correlated with visual impairment. Hyperreflective foci in the outer retinal layers on SD-OCT images, another marker of visual disturbance, are associated with foveal photoreceptor damage.

## 1. Introduction

Diabetic macular edema (DME), a leading cause of visual impairment in individuals of working age, is mediated by multiple and complex mechanisms in its pathogenesis [1–4]. Pathophysiology, that is, vascular hyperpermeability and ischemia, is represented by clinical findings seen on fluorescein angiography (FA) images [5–8]. Basic research has elucidated the molecular mechanisms including vascular endothelial growth factor in DME and proliferative diabetic retinopathy (PDR) [9–18]. Using biomicroscopy, clinical ophthalmologists observe thickening of the sensory retina and vascular lesions in DME. However, it is difficult to objectively evaluate the neuroglial changes in the retina. In contrast, histologic studies have reported that cystoid spaces are present mainly in the inner nuclear layer (INL) and the outer plexiform layer (OPL) and contribute partly to macular thickening [19–21]. Electron microscopy showed that, in addition to the accumulation of the extracellular fluids, intracytoplasmic swelling of the Müller cells might be a pathophysiologic mechanism in DME. 

**Figure 1:**
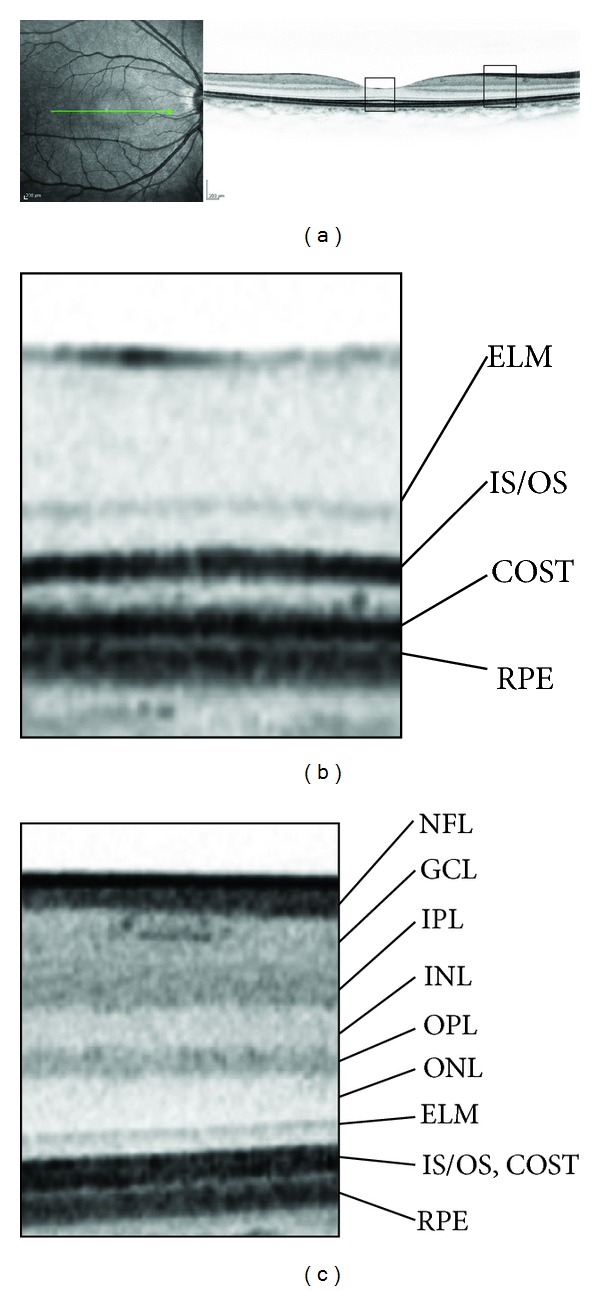
(a) Retinal sections of the physiologic macula dissecting along the green line on fundus photography using SD-OCT. (b) The magnified image at the fovea shows three lines over the retinal pigment epithelium (RPE), that is, the external limiting membrane (ELM), the junction between inner and outer segments (IS/OS), and the cone outer segment tips (COST). (c) A magnified parafoveal image shows the individual retinal layers. NFL: nerve fiber layer; GCL: ganglion cell layer; IPL: inner plexiform layer; INL: inner nuclear layer; OPL: outer plexiform layer; ONL: outer nuclear layer.

Optical coherence tomography (OCT) provides retinal sectional images as in histology study (Figure 1) and is useful for qualitative and quantitative evaluation of pathological retinal changes [22]. The original instrument, time-domain OCT, has been replaced with spectral-domain OCT (SD-OCT), which has higher resolution and reduced speckle noise. Clinicians can appreciate the improved delineation of the fine pathological lesions and the clearer borders between the individual retinal layers. 

This review summarizes the current understanding of the retinal thickness, pathomorphologies, and photoreceptor damage in DME that can be seen on OCT. 

## 2. Retinal Thickening on OCT Images

**Figure 2:**
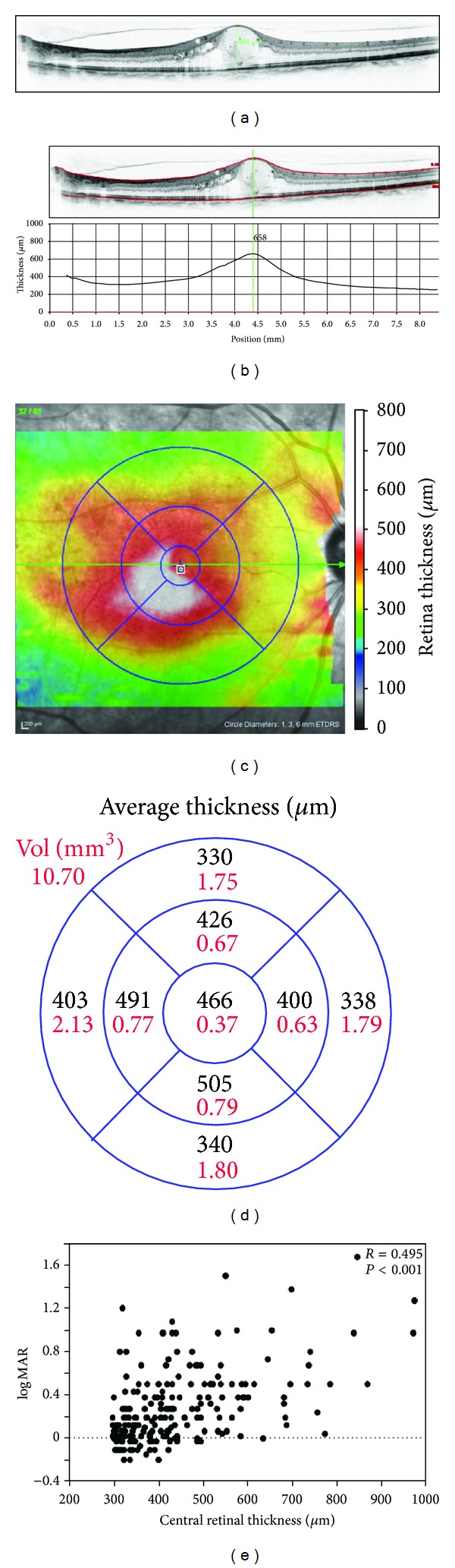
Quantification of the total retinal thickness using OCT. The retinal thickness can be measured semiautomatically (b) or manually using calipers (a). (c) A two-dimensional map of the retinal thickness can be constructed from the automatically measured retinal thickness. (d) The average thickness in each subfield of Early Treatment Diabetic Retinopathy Study grid is shown. (e) The average thickness in the central subfield is correlated modestly with the logarithm of the minimum angle of resolution VA in center-involved DME.

Biomicroscopy allows ophthalmologists to subjectively evaluate retinal thickening, whereas OCT has enabled objective quantification of the total retinal thickness (Figure 2). The diabetic retinopathy clinical research network (DRCRnet) especially defined an increase in the mean central thickness of 1 mm as center-involved DME [23, 24], which is the new standard for applying treatments and a surrogate marker for evaluating treatment efficacy [25]. It is widely accepted that the central thickness is correlated modestly with visual acuity (VA) in DME [26]. In addition, the DRCRnet reported that paradoxical VA changes are observed after intervention, that is, VA reduction despite resolution of ME or VA improvement with increased retinal thickening [26]. These data suggest the importance of identifying unknown mechanisms and the magnitude of edematous changes. Despite these issues, an increasing number of studies have reported the relevance of measuring the retinal thickness after treatment for DME as a surrogate marker [27–29].

SD-OCT with higher resolution and reduced speckle noises has enabled segmentation of the individual retinal layers, and several kinds of OCT instruments provide automated segmentation. This feature has encouraged quantification of the thickness of the retinal layers and qualitative evaluation of lesions in the individual layers. Interestingly, the thickness in the inner retinal layers is correlated positively with visual impairment, whereas the outer retinal thickness is associated negatively with poor visual prognosis after vitrectomy for DME [30]. This suggests that thinning of the outer retinal layers is related to photoreceptor degeneration (or atrophy) concomitantly contributes to visual disturbance at least partly, and supports the paradoxical VA changes reported by the DRCRnet [26]. Further analyses of retinal thicknesses with segmentation would improve the understanding of the association between clinical findings and pathogenesis in DME.

## 3. Pathomorphology in Individual Retinal Layers

Numerous pathological mechanisms have been reported regarding diabetic retinopathy (DR), compared to the simple criteria of diabetes per se [1–4]. OCT subjectively shows several types of foveal morphologies in DME, that is, cystoid macular edema (CME), serous retinal detachment (SRD), and sponge-like retinal swelling [31], which might be among other factors that modulate visual function, dependently or independently of retinal thickening. Individual lesions have been delineated in the individual retinal layers. OCT showed cystoid spaces mainly in the INL and OPL, which has been supported by histologic reports [20, 32, 33]. Extracellular fluids pool between the outer segments and retinal pigment epithelium (RPE) in eyes with SRD. Sponge-like retinal swelling at the fovea occurs in the OPL. 

**Figure 3:**
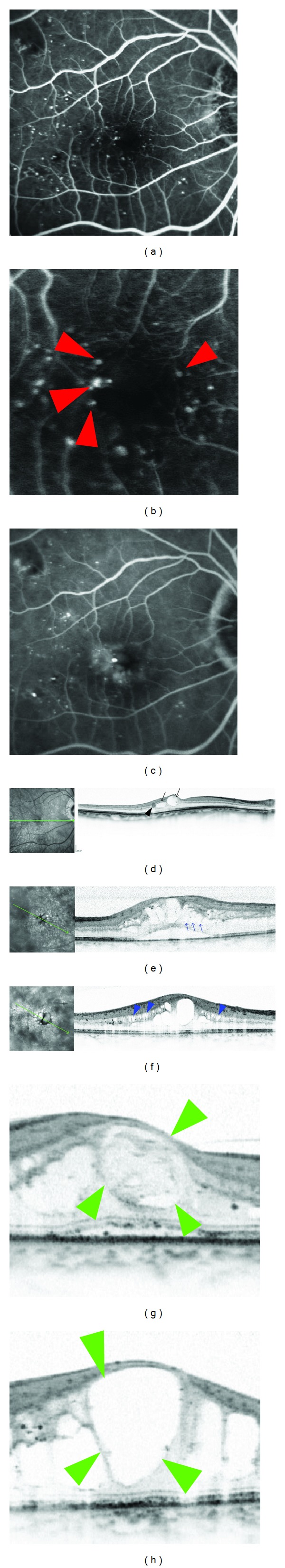
Characteristics of cystoid spaces in DME. ((a), (b)) Early-phase FA shows an enlarged foveal avascular zone and surrounding microaneurysms (arrowheads). (c) A late-phase image shows fluorescein pooling at the fovea. (d) Cystoid spaces (arrows) are accompanied by microaneurysms (arrowhead). (e) A late-phase FA image shows petalloid- (e) or honeycomb-pattern (f) fluorescein pooling, which is considered to correspond to cystoid spaces in OPL (arrow) or INL (arrowhead), respectively, on OCT image. Foveal cystoid spaces (arrowheads) have higher OCT reflectivity and its heterogeneity (g) or lower and homogeneous reflectivity (h).

Regarding the types of CME, OCT has documented several findings (Figure 3). The cystoid spaces in the INL have a honeycomb pattern of fluorescein pooling, whereas petalloid-shaped pooling corresponds to cystoid spaces in the OPL [32, 33]. Further study of an FA/OCT correlation found that the foveal cystoid spaces on OCT images were associated with enlarged foveal avascular zones and microaneurysms around the perifoveal capillary network [8]. This suggested that ischemia and leakage from microaneurysms contribute to the development or maintenance of cystoid spaces in DME. A recent publication reported that OCT reflectivity in the cystoid spaces was correlated negatively with the intensity of the fluorescein pooling [34] and implicated several types of vascular hyperpermeability regarding the pathogenesis in the cystoid spaces.

SD-OCT has shown the fine structures of the microaneurysms in patients with DR [35–37]. Among the capsular structure patterns, the “incomplete” and “absent” types, in contrast to the “complete” type, often were accompanied by fluorescein leakage and cystoid spaces [36, 37]. Recently, using SD-OCT, clinicians have confirmed that the number of microaneurysms decrease after focal photocoagulation, which suggested that in the future FA would be replaced with noninvasive OCT to evaluate treatment efficacy [38, 39].

Vitreomacular traction sometimes modulates macular thickening in DME [40–43]. Eliminating the vitreoretinal traction during vitrectomy is suggested to be an effective strategy for eyes with DME, and DRCRnet has reported the greater VA improvement in eyes with preoperative ERM and the greater reduction of central subfield thickness in eyes with vitreoretinal abnormalities [44–46]. Ocriplasmin, a recombinant protease with activity against components of the vitreoretinal interface, has recently been reported to be effective for the diseases with vitreomacular interface abnormalities [47]. It remains to be elucidated how ocriplasmin modulates retinal thickening without the removal of the vitreous gel which contains growth factors and cytokines. 

**Figure 4:**
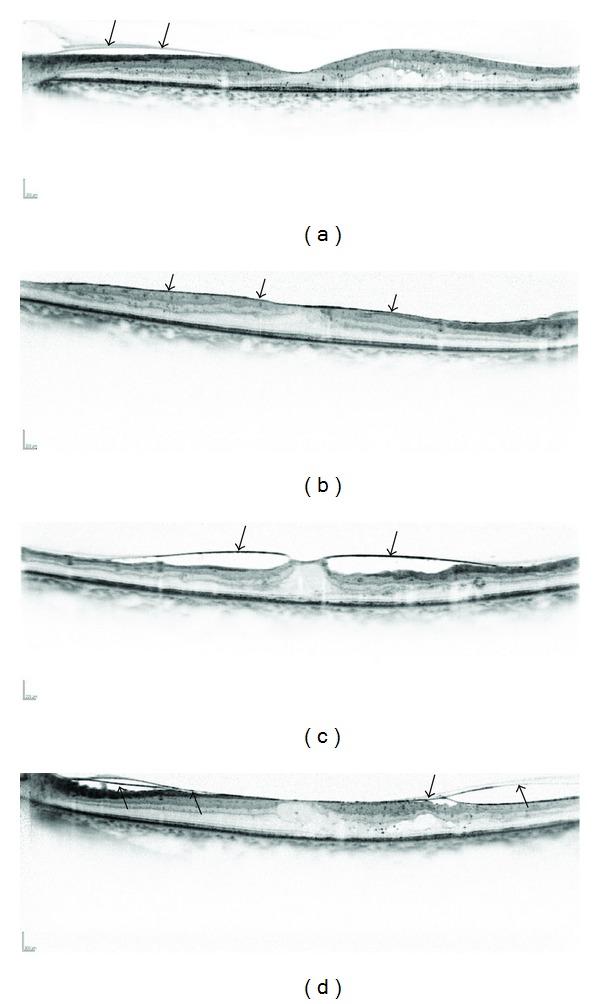
Several patterns of vitreomacular interface abnormalities in DME. (a) A posterior hyaloid membrane is sometimes depicted on OCT images (arrows). (b) ERM often is accompanied by sponge-like retinal swelling (arrows). (c) A perifoveal vitreous separation sometimes induces vertical traction, as in idiopathic macular holes (arrows). (d) Some eyes have separation of a posterior vitreous membrane (arrows).

Sponge-like retinal swelling especially often is accompanied by pathological findings in the vitreomacular interface on OCT images (Figure 4), and vitreomacular interface abnormalities contribute to thickening of the retinal parenchyma in the OPL at the fovea, as in the case of idiopathic epiretinal membrane (ERM). It also was reported that vitreomacular interface abnormalities also might induce SRD in DME [40], and OCT sometimes shows cystoid spaces with vitreoretinal abnormalities as with idiopathic macular holes [48, 49]. Fibrovascular proliferation in PDR progresses along the posterior hyaloid membrane and induces contraction. Tangential tractional forces increase the retinal thickness and concomitantly contribute to macular edema, and horizontal forces result in macular heterotopia (traction maculopathy) in PDR. Several patterns of vitreomacular interface abnormalities seen on SD-OCT images were reported recently, that is, vitreomacular traction with no or partial posterior vitreous detachment, posterior vitreous separation, ERM, and their combinations. These findings would help surgeons to completely remove the pathological changes of the vitreoretinal interface [43].

**Figure 5:**
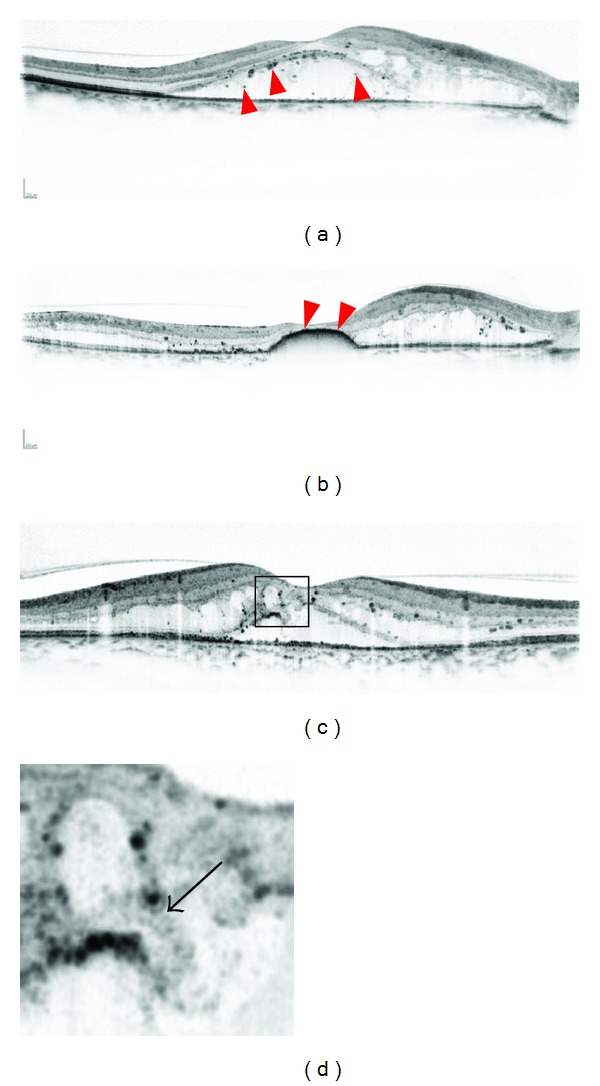
A representative case with hyperreflective foci (arrowheads) in subretinal fluids. The preoperative decimal VA is 0.6. (b) DME is improving after focal/grid photocoagulation, although hyperreflective foci have accumulated at the fovea (arrowheads). The postoperative VA is 0.09. (c) OCT sometimes shows that cystoid spaces in the OPL rupture to subretinal fluids (arrow), which might modulate the pathogenesis in SRD.

It is not well known how SRD develops in DME compared to the pathogenesis in eyes with CME or sponge-like retinal swelling. Marmor reported numerous clinical and basic data regarding the development of retinal detachment that depended on the osmotic or oncotic pressure of intraocular fluids [50]. In eyes with DME, vascular hyperpermeability might increase such pressures, resulting in SRD. High-resolution OCT has enabled observation of the cystoid spaces in the OPL that sometimes rupture toward the SRD, suggesting that extravasated blood components pour directly into the SRD [51]. Regarding visual function, no association was found between VA and foveal thickness in eyes with foveal SRD [52], whereas these eyes often have a poor prognosis after treatment [51, 53]. OCT often delineates hyperreflective foci in subretinal fluids (Figure 5). In most such cases, subfoveal hard exudates develop after resolution of the macular edema (ME) that correspond to the confluent hyperreflective foci with concomitant impaired visual function [51], as reported in the Early Treatment Diabetic Retinopathy Study [54, 55]. 

## 4. Photoreceptor Layers

**Figure 6:**
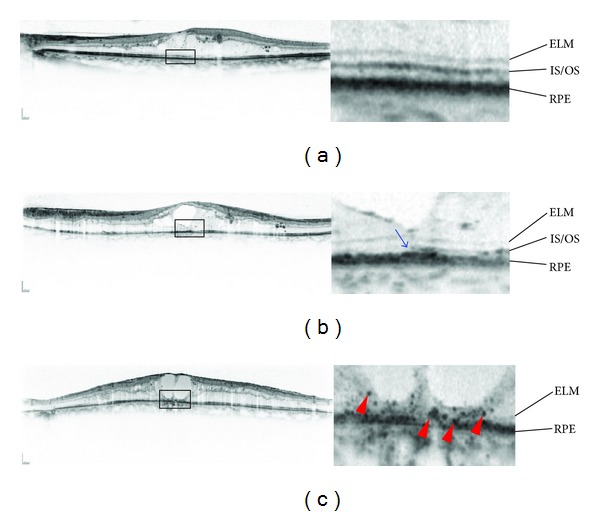
Pathological changes in the photoreceptor layers in DME. (a) Both the ELM and IS/OS lines are continuous. (b) The ELM line seems almost continuous, whereas the IS/OS line is discontinuous on the left (arrow). (c) The ELM line is disrupted, and the IS/OS line is absent at the fovea, accompanied by hyperreflective foci in the outer retinal layers (arrowheads).

The superior delineation of the fine structures on SD-OCT images encouraged us to evaluate photoreceptor markers, external limiting membrane (ELM), and the junction between the inner and outer segments (IS/OS) (Figure 6). Sandberg and associates reported that the IS/OS line, to which they referred as the third high-reflectance band in their original manuscript, is associated with visual function in retinitis pigmentosa [56], suggesting that the IS/OS line represents the photoreceptor structure and function *in vivo* [57]. A few years later, disruption of the IS/OS line at the fovea was reported to be associated with a poor visual prognosis in resolved ME associated with branch retinal vein occlusion [58]. Many later cross-sectional or longitudinal studies have shown the clinical relevance of the IS/OS line in DME [59–67]. Histologic publications have reported “cystoid degeneration” in the photoreceptors at the fovea [68], which supports the disruption of the IS/OS line on OCT images. The thickness of the photoreceptor outer segments was quantified and found to be associated with visual function in DME [62]. The transverse length of the disrupted or absent IS/OS line also has been related to visual impairment [63, 66]. In the future, quantification of photoreceptor damage would improve the understanding of visual impairment in DME.

Despite the relevance, it is unknown whether the IS/OS line seen on OCT images truly corresponds to the histologic junction of the inner and outer segments. Spaide and Curcio speculated that this highly reflective band was located at the ellipsoid in the inner segments, considering the correlation between the microstructure on the SD-OCT images and the histologic findings [69]. The OCT reflectivity changed around the line after light exposure, which suggested that the line may represent photoreceptor function per se [70, 71]. 

 The ELM line is another marker of photoreceptor integrity, and its disruption also is associated with visual impairment in DME [52, 66, 67]. Shin and associates reported that ELM disruption predicts poor visual outcomes after treatment with triamcinolone [67]. Since the ELM is an intercellular junction between the Müller cells and photoreceptor cells and has barrier properties against macromolecules [72], the disrupted ELM might allow blood components to migrate into the outer retinal layers and exacerbate the photoreceptor damage. Although it remains poorly understood how the ELM becomes disrupted, a few possible mechanisms are implicated. Extended cystoid spaces from the INL to the OPL are accompanied by ELM disruption in DME, suggesting disturbance of the Müller cells [66]. A tear in the outer retinal layers also can result in loss of the barrier function in eyes with SRD [51]. 

SD-OCT shows dot-like lesions, referred to as hyperreflective foci, throughout the retina in DR [35]. Those in the outer layers especially are correlated cross-sectionally with poor visual function in patients with DME without SRD [73]. Hyperreflective foci in subretinal fluids accumulate at the fovea and lead to poor visual prognosis in eyes with SRD [51]. Bolz and associates speculated that the hyperreflective foci in DR corresponded to lipid-laden macrophages and the precursors of hard exudates [35]. The hyperreflective foci also are considered to be degenerated photoreceptors and RPE hyperplasia or metaplasia in other diseases [74, 75]. Although it remains undetermined what the hyperreflective foci are in the outer retinal layers in DME, the disruption of the ELM or IS/OS line is correlated with the hyperreflective foci, suggesting that these lesions reciprocally promote the pathogenesis of photoreceptor degeneration.

## 5. Ganglion Cells and Nerve Fiber Layers

The nerve fiber layer (NFL) is comprised of axons derived from ganglion cells. The defects in the NFL were clinically reported [76], and basic research showed ganglion cell apoptosis in DR [77]. Since swelling of the NFL occurs in lesions associated with DR, such as cotton-wool spots (soft exudates), it is difficult to evaluate the decrease in the axons from the ganglion cells using OCT. In contrast, thinning of the ganglion cell layer was reported in eyes with ischemic maculopathy with and without DME [78]. Further, glaucoma research has focused on the clinical relevance of the ganglion cell complex (from the inner limiting membrane to the outer boundary of the inner plexiform layer) [79], which should be applied to evaluate neovascular glaucoma in advanced PDR. 

It was reported that white spots on fundus photography might correspond to hyperreflective lesions at the level of NFL [80]. Midena and associates have recently demonstrated that hyperreflective spots, which might correspond to activated microglia or Müller cells, were detected in inner retinal layers, as DR progresses. They were suggested to be a novel biomarker of glial activation in DR, and further investigation remains to be planned [81]. 

## 6. The Choroid

Disruption of the choroidal circulation in patients with diabetes had been reported as diabetic choroidopathy [82, 83]. Enhanced-depth imaging using SD-OCT or the latest version of OCT, swept-source OCT, recently has enabled measurement of the choroidal thickness. A few publications have reported alteration of the choroidal thickness [84–86] and further investigations will clarify how the pathological choroidal changes contribute to DME.

## 7. Conclusions

OCT has allowed identification of the morphologic factors in the pathogenesis in DME. The major OCT parameter, the central retinal thickness, is correlated modestly with the VA, and pathomorphologies and photoreceptor damage also cause visual impairment. Further studies of a structural-functional correlation will promote a better understanding of the complex pathogenesis in DME [87].

## Abbreviations

CME: Cystoid macular edema
DME: Diabetic macular edema
DR: Diabetic retinopathy
ELM: External limiting membrane
ERM: Epiretinal membrane
FA: Fluorescein angiography
INL: Inner nuclear layer
IS/OS: The junction between inner and outer segments
ME: Macular edema
OPL: Outer plexiform layer
PDR: Proliferative diabetic retinopathy
SD-OCT: Spectral-domain optical coherence tomography
SRD: Serous retinal detachment.
